# Smaller Feedback-Related Negativity (FRN) Reflects the Risky Decision-Making Deficits of Methamphetamine Dependent Individuals

**DOI:** 10.3389/fpsyt.2020.00320

**Published:** 2020-04-21

**Authors:** Na Zhong, Tianzhen Chen, Youwei Zhu, Hang Su, Xiaolu Ruan, Xiaotong Li, Haoye Tan, Haifeng Jiang, Jiang Du, Min Zhao

**Affiliations:** ^1^Shanghai Mental Health Center, Shanghai Jiaotong University School of Medicine, Shanghai, China; ^2^Shanghai Key Laboratory of Psychotic Disorders, Shanghai, China

**Keywords:** methamphetamine, dependence, risky decision-making, feedback-related negativity (FRN), impulsivity

## Abstract

Methamphetamine (MA) chronic users show risky decision-making deficits. However, the neural mechanisms underlying these deficits remain unclear. A case-control study was conducted to understand how MA users and healthy controls differ in electrophysiological responses associated with series decision-making. Electroencephalography of 31 MA users and 27 healthy controls was recorded when they performed the Balloon Analogue Risk Task involving risky decision-making with uncertain gain or loss. Feedback-related negativity (FRN) was measured and their association with their risky decision-making and impulsivity were examined. Compared to healthy controls, MA users showed smaller peak FRN amplitudes in fronto-central electrodes (F _(1,__56)_ =4.559, p=0.037), and the attenuated peak FRN amplitudes correlated with more risk-taking behavior (r=0.48, p=0.012). Besides, MA users exhibited later FRN (F _(1,__56)_ = 7.561, p=0.008) and earlier P300 (F _(1,__56)_ = 3.582, p = 0.041) compared to healthy controls in fronto-central electrodes, which were correlated with higher score of impulsivity. These findings provided further evidence that MA users showed insensitivity to negative feedback in risky decision-making. FRN might be a promising biomarker of dependence.

## Introduction

Methamphetamine (MA) is one of the most used substances, which has caused severe physical or psychological negative consequences to its chronic users (1, 2). Chronic MA use showed risky decision-making deficits (3–5), and these risky decision-making deficits could likely be a premorbid factors or could be induced by substance use (6–8). Previous studies proved that the poor risky decision-making ability of MA users could aggravate the appearance of negative consequences, such as the higher risk of relapse, lower adherence (3, 6, 9). Thus, better understanding the risky decision-making deficits in chronic MA users might find effective treatment approaches or relapse predictors.

Electroencephalography (EEG) recordings during the performing risky decision-making task provided an opportunity to know better about the underpinning of chronic MA users ‘decision-making deficits. The feedback-related negativity (FRN) is an electrical brain signal that usually peaks 250 ms after unfavorable feedback and showed at frontal-central region, and it most likely generated from the anterior cingulate cortex (ACC) when individuals received the negative feedback of their performance and reflected a prediction error (10–12). And some researchers considered the FRN as one kind of error-related negativity, although ERN usually peaks 100ms after error while FRN peaks at 250ms after feedback (10, 13, 14). Moreover, the FRN is associated with the functional connection between the medial frontal cortex and the prefrontal cortex (15). Besides, the P300 that peaks 300–600 ms after feedback presentation is associated with the later, attention-sensitive cognitive processing to the feedback, such as information processing, emotional, or reward processing (16). And P300 amplitude is considered to relate to reward valence, reward magnitude, which generated by the ACC and others circuit among frontal areas as well (17, 18).

Studies for the effects of chronic MA use to neural responses to feedback are limited and inconsistent. Two studies showed that MA users performed worse in risky decision tasks, and showed less sensitivity to risk in DLPFC, ventral striatum, striatum, and post caudate than healthy controls by functional magnetic resonance imaging (fMRI) (5, 19). A study showed that female MA users showed enhanced FRN for monetary losses after the anticipatory reward stage in a simple gambling task compared to healthy controls, and the study just recruited female MA users with averaged 9-months abstinent by EEG (20). However, several previous studies proved that patients with chronic alcohol use, chronic cocaine use, gambling disorder, and problematic Internet behaviors showed decreased neural responses to negative feedback in risky decision-making tasks by EEG (16, 21–23). The neural responses of chronic MA users to unfavorable outcome during the risky decision-making and error process is unclear and inconsistent, it needs more studies to understand the neural mechanism of the risky decision-making in this large population.

The balloon analogue risk task (BART) is a good approach to test risk-taking behavior and understand the neural mechanism of risky decision-making for MA patients (24, 25). BART required individuals to balance the loss and reward like in the real world risk decision-making situation, through continuously pumping a balloon to gains earnings while risking it or stop pumping and collect the current earnings (24). Previous studies found that smokers, alcohol users, gamblers, 3,4-methylenedioxy-methamphetamine users showed more pumps than non-drug users in BART tasks, and the BART performance was correlated higher impulsivity traits (24–26). The neural response of chronic MA users during preforming BART is unknown, while understanding that would help us find the brain mechanisms involved in the process of negative feedback.

And previous studies showed the impulsive personal traits were tightly related with risky decision-making and the feedback processing (24), and the cognitive deficits or impulsive personality traits was endophenotypes or drug-induced neurotoxicity (27, 28). More studies are needed to understand the impact of the impulsive personal traits on the neural responses to negative feedback in risky decision-making.

In this study, the event-related brain potentials (ERPs) during the risky decision-making task between MA users and healthy controls were tested, and impulsivity traits, cognitive function as well, to understand the neural mechanism of risky decision-making in MA dependence and its related factors. We hypothesized that the chronic MA users showed poor risky decision-making ability, and display neural insensitivity to unfavorable feedback represented by smaller FRN and P300 potentials, which is associated with high impulsive traits compared to healthy controls.

## Method

### Participants

Two groups were recruited: 31 MA chronic users from Shanghai Compulsory Rehabilitation Center, and 27 age, gender-matched healthy controls. The inclusion and exclusion criteria included: (1) between 18–60 years old; (2) Han nationality; (3) MA users should meet diagnosis criteria of MA dependence based on Diagnostic and Statistical Manual of Mental Disorders criteria (DSM-IV); (4) without severe cognitive impairments; (5) without other present and history axis I psychiatric disorders in DSM-IV, except for the methamphetamine induced psychosis in the past; (3) without other substance abuse or dependence except for nicotine.

Shanghai Mental Health Center (permission number: 2016-11R) approved and monitored the study to ensure that the study met the ethical requirement. All subjects were voluntary and gave written informed consent, moreover, they could quit the study freely at any moments and their decision would not impact their normal rehabilitation activities.

### Measurements

Balloon analog risk task (BART) was a computerized cognitive tool for risky decision-making (24). The participants were asked to gain as much virtual money as they can by continuously inflating a balloon showing on the screen (positive feedback) while risking losing current virtual money when the balloon was burst (negative feedback), or stopping inflating the balloon and collecting the current virtual money. There were 100 trials for each participant (i.e., 100 balloons) and the random probability of each balloon explosion was 1–12 pumps. Participants did not have a time limit to continuously pump the balloon or collect the current virtual money. In each trial between feedback stimulus and participant's corresponding reaction, a 1,000–1,500 ms random delay was introduced. If the balloon exposed or been collected, the pictures of a burst balloon with zero or inflated balloon with current virtual money were presented for 2,000 ms. The scores of BART were adjusted averaged number of pumps on unexploded balloons, the number of balloon bursts (24).

Barratt Impulsiveness Scale version 11 (BIS -11) is a 30-item, self-report scale widely used to test impulsivity trait, and its Chinese version shows good reliability and validity (29), including subsets of motor impulsiveness, cognitive impulsiveness, and non-planning impulsiveness (30). The score for each item is from 1–4, with higher scores representing higher impulsivity.

The CogState Battery is a computerized cognitive test with good reliability and validity (31), and we applied five tasks from it, including the Two Back Task (TWOB) for working memory, International Shopping List Task (ISL) for verbal learning and memory, the Groton Maze Learning Task (GML) for problem-solving, Social-Emotional Cognition Task (SEC), and the Continuous Paired Association Learning Task (CPAL) for spatial working memory. Detailed procedures of these tasks have been described elsewhere (27).

### EEG Recording and Processing

#### EEG Recording

While participants performed the BART continuous EEG data were recorded from a high-density 64-channel electrodes cap (BrainCap; Asiacut, Germany) with online signal filtering (0.1–200 Hz), and sampling frequency was 1,000 HZ with all impedances below 5 kΩ. We measured the vertical electrooculography (EOG) on the up and down the right eye, the horizontal EOG on the outer of both eyes, the reference electrode at the tip of the nose, and the ground electrode on the forehead.

#### EEG Processing

EEG data was preprocessed by EEGLAB (Version: 13.5.4b) as well as ERPLAB (Version: 6.0) toolboxes based on Matlab (Version: R2014a) (32, 33). The offline band-pass filter (0.1–40Hz) and a notch filter (50 Hz) were used. Independent component analysis (ICA) was applied to modify eye movement or heartbeat effect. Epochs were extracted from -200 to 800ms after the feedback, and averaged EEG data from -200ms to 0ms was used as the baseline. And all EEG epochs were processed for artifact detection by ERPLAB, including detection for maximal 150 μV-threshold amplitude difference within a 200 ms-width and 50 ms-step moving window, detection for maximal ± 100 μV-threshold absolute amplitude, and examination of obvious eye movement or eye blinks. The range of the negative feedback-locked waveforms for each subject was 23–66 based.

### Procedures

Thirty-one MA chronic users and 27 healthy controls were recruited from June 2017 to August 2018, and after the informed consent, all the subjects finished a 60 min face-to-face interview for their demographic data, drug use histories, the severity of drug use, and impulsivity trait (BIS-11). On the other day, the subjects were asked to complete the 30 min cognitive function tests (the Cogstate Battery) and performed 25 min risky-decision making task (the BART task) and their continuous EEG data were recorded at the same time. The subjects were encouraged to finish all tests continuously, while they could have short breaks to avoid fatigue. To avoid the impact on cognitive tests from fatigue or nicotine abstinence, the participants were allowed to have approximately 5-min short breaks and had cigarettes 30 min before the cognitive test.

### Statistical Analysis

All the data were processed by Statistical Package for the Social Sciences (SPSS), including *t*-tests were for continuous variables comparison, and Chi-square tests for dichotomous variables comparison, and multivariate analyses of variance (MANOVA) for overall cognitive function comparison.

For the ERP analysis, feedback-locked epochs including positive and negative feedbacks were calculated separately, and difference waves were produced by subtracting the positive feedback waves from the negative feedback waves (23). Then, we measured the peak amplitudes and latencies of FRN in the 200–300 ms period and P300 in the 300–600 ms period. The ERPs data met the Normal distribution. Since the neural responses to feedback in risky decision-making were associated with activation of ACC and prefrontal cortex and previous ERP studies with the BART used fronto-central electrodes (15, 16, 21–23), the fronto-central electrodes of Fz, FCz, Cz where the FRN and P300 were most obvious were chosen for analysis, and 2*3 repeated ANOVAs with groups as a between-subjects factor and electrodes as a within-subjects factor were used, followed by *t*-tests when appropriate. Pearson correlation was used to find the relationship between brain potentials and risk-taking behavior, drug use histories, cognitive function, and impulsive traits. The significant level was 0.05.

## Results

### Demographic and Drug Use Features

The averaged years of MA use was 7.16 (SD=5.64) for the chronic MA users, and at the assessment point, the averaged days of abstinence was 76.35 (SD=43.36). Except for the years of education and the proportion of smoking, there were no group differences between MA users and healthy controls (**Table 1**)

**Table 1:** Demographic and drug use features of methamphetamine (MA) users and controls.

	MA users (n=31)	Health controls (n=27)	*t* or *χ^2^ value*	P value
Age^a^	33.48(6.90)	30.96(7.18)	0.96	0.342
Female^b^	15(48.4%)	9(33.3%)	1.35	0.246
Years of education^a^*	10.03(2.49)	13.74(3.16)	5.00	<0.001
Married, n (%)^b^	22(71.0%)	15(55.6%)	1.46	0.278
Alcohol use, n (%)^b^	19(61.3%)	12(44.4%)	1.65	0.200
Smoking, n (%)^b^*	27(87.1%)	11(40.7%)	13.73	<0.001
MA onset age	25.68(8.20)			
Years of MA use	7.16(5.64)			
Days of abstinence	76.35(43.36)			
Marijuana use, n (%)	8(25.8%)			

The data were the mean (SD, standard deviation) or number (proportion). ^a^t-Test, ^b^χ^2^ test, *p < 0.05.

### The Risky Decision-Making and Clinical Features Between the Two Groups

The scores of BART and impulsiveness between two groups were analyzed by *t-*tests, there was a trend in the difference of the Number of burst balloons (*t*=1.92, p=0.052) and a significant difference in averaged decision time for pumps (*t*=2.05, p=0.045). Besides, there were significant differences in the total scores of BIS (t=5.13, p<0.001) and all its subscales (p<0.05) between MA and HC groups.

MANOVA compared the performance of the cognitive tests between MA and HC groups, and there was a significant difference the whole cognitive test (Wilk's λ=0.79, p=0.049), followed *t*-tests showed that MA group performed worse on ISL task (*t*=2.31, p=0.025) and CPAL task (*t*=2.73, p=0.022) compared to Healthy controls (**Table 2**)

**Table 2:** Risky decision-making and clinical features of methamphetamine (MA) users and controls.

	MA users (n=31)	Health controls (n=27)	*t* value		P value
BART total score	2405.44(354.23)	2498.88(272.57)	1.23		0.221
Number of burst balloons*	52.76(12.45)	47.48(11.06)	1.92		0.052
Adjusted averaged pumps	4.96(1.73)	4.99(1.19)	0.10		0.925
Decision-making time*	439.77(153.09)	532.75(192.02)	2.05		0.045
BIS Total score*	84.35(16.99)	65.15(11.04)	5.13		<0.001
Motor impulsiveness*	27.09(7.99)	21.85(3.91)	3.17		0.002
Cognitive impulsiveness*	27.29(7.57)	21.70(4.50)	3.35		0.001
No planning impulsiveness*	30.29(8.51)	21.70(5.51)	4.62		<0.001
Cogstate battery					
TWOB (accuracy rate)	1.02(0.22)	0.96 (0.22)	1.03	0.307	
GML (total error)	66.00(19.19)	56.27 (45.42)	0.03	0.495	
ISL (total correct) *	19.37(5.49)	23.00(6.04)	2.31	0.025	
SEC (accuracy rate)	0.963(0.23)	0.94(0.23)	0.39	0.695	
CPAL (total error) *	93.07(51.11)	57.72(55.61)	2.37	0.022	

The data were the mean (SD, standard deviation). *P<0.05. BART, balloon analogue risk task; BIS, Barratt impulsiveness scale; CPAL, continuous paired association task; GML, Groton maze learning task; ISL, international shopping list task; SEC, social emotional cognition; TWOB, two-back task.

### Event-Related Brain Potentials Between MA Addicts and Healthy Controls

**Figure 1:**
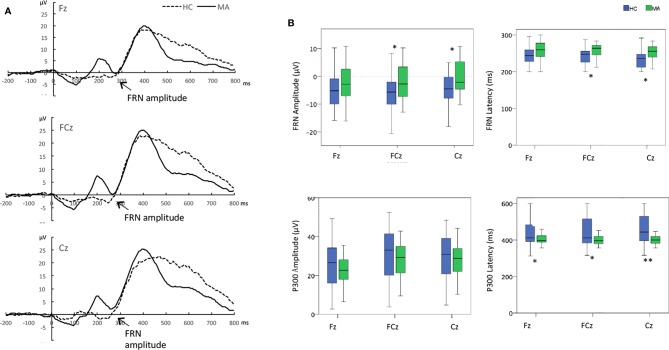
**(A)** Overall event-related brain potential (ERP) difference waves for the methamphetamine (MA) users and healthy controls. **(B)** The difference between MA users and healthy controls the amplitudes and latency at electrodes of Fz, FCz, and Cz. *p < 0.05 **p < 0.01, t-tests between groups at each electrode when group effect was significant. Time zero is the presentation of feedback.

Grand averaged ERP difference for FRN and P300 were shown in **Figure 1A**. And the The measurement data of FRN amplitude and latency were analyzed by 2 (groups)*3(electrodes, Fz, FCz, Cz) ANOVAs. FRN amplitude: The significant group effect (F _(1,56)_=4.56, p=0.037) and the electrode effect (F _(2,112)_=5.86, p=0.012) were found, but without significant group*electrode effect in FRN amplitude. The FRN amplitudes in FCz electrode (-2.73 μV Vs -5.77 μV) and Cz electrode (-0.10 μV Vs -4.54 μV) were significant less negative in the MA group than in the HC group (P<0.05). FRN latency: The significant group effect was found (F _(1,__56)_ = 7.56, p=0.008) in FRN latency but without significant electrodes and group*electronic interaction effects. The FRN latency in FCz electrode (258.97 ms Vs 242.96 ms) and Cz electrode (254.06 ms Vs 236.74 ms) significantly showed later in the MA group than in the HC group by *t*-test (P<0.05) (**Table 3**, **Figure 1B**).

**Table 3:** The peak amplitude and latency of the feedback-related negativity (FRN) and P300 in event-related brain potential (ERP) difference waves in frontal-central electrodes in the methamphetamine (MA) group and HC group.

	MA group			HC group			Group effects		Electrode effect		Group*electrode	
	Fz	FCz	Cz	Fz	FCz	Cz	F	p	F	p	F	p
FRN amplitude (μV)	-2.73 (6.77)	-2.73 (6.68)	-0.10 (6.23)	-4.81 (6.83)	-5.77 (6.90)	-4.54 (5.66)	4.56	**0.037**	5.86	**0.012**	3.00	0.054
FRN latency (ms)	258.19 (26.71)	258.97 (19.07)	254.06 (20.21)	246.22 (29.32)	242.96 (26.81)	236.74 (12.38)	7.56	**0.008**	2.51	0.102	0.37	0.625
P300 amplitude (μV)	22.69 (7.74)	27.93 (8.67)	27.87 (8.14)	25.45 (12.39)	31.04 (14.03)	28.96 (12.21)	0.72	0.400	48.03	**<0.01**	1.68	0.19
P300 latency (ms)	405.80 (32.51)	401.80 (27.32)	402.83 (26.17)	439.41 (71.87)	448.15 (78.15)	459.70 (78.99)	10.21	**0.002**	3.53	**0.041**	6.06	**0.006**

The data are the mean (SD).

Bold values indicate significance at P < 0.05 compared with the HC group.

The measurement data of P300 amplitude and latency were analyzed by the same ANOVAs. P300 amplitude: There was only a significant electrodes effect (F _(2,112)_= 48.03, p<0.01) but without significant group or group*electrode interaction effects. The P300 amplitudes at FCz and Cz electrodes were larger than that at Fz electrode in all subjects (P<0.05). P300 latency: There were significant group effect (F _(1,__56)_ = 10.21, p < 0.01), as well as group*electrode interaction effect (F _(2,__112)_ = 6.06, p < 0.01) and electrodes (F _(2,__112)_ = 3.58, p = 0.041). Post-hoc analysis revealed that the P300 latency of MA users was earlier than that in healthy controls at the electrodes of Fz, FCz, and Cz (P<0.05) (**Table 3**, **Figure 1B**).

### The Correlations Between Event-Related Potentials and Risky-Decision Making

The averaged peak FRN amplitudes from the frontal-central electrodes were significantly associated with the total number of burst balloons (r=0.31, P=0.017) and also the scores of the BIS (r=0.30, P=0.021) for all the participants, which indicated that individuals with smaller FRN amplitude had more burst balloons and higher BIS score. Moreover, the averaged peak FRN latency was related to the total score of BIS (r=0.28, p=0.036) across, and the averaged peak P300 latency was negatively related with the total score of BIS(r=-0.30, p=0.022) across all the subjects (**Table 4**).

**Table 4:** Relationship between event-related brain potentials (ERPs) and risky decision-making, impulsivity, and clinical features within individual and whole groups.

	FRN		P300	
	Amplitude (μV)	Latency (ms)	Amplitude (μV)	Latency (ms)
**BART (MA group)**				
Total score	-0.027	0.032	-0.115	-0.240
Number of burst balloons	0.477*	0.349	0.284	-0.050
Averaged pumps	0.304	0.230	0.157	-0.031
Decision-making time	0.091	0.002	0.307	0.084
BIS (MA)	0.219	0.143	0.032	0.018
Years of MA use	-0.219	-0.080	-0.266	0.189
Days of abstinence	0.088	0.123	0.196	-0.243
**BART (HC group)**				
Total score	-0.031	-0.181	0.118	-0.204
Number of burst balloons	0.163	0.104	0.047	-0.188
Averaged pumps	0.112	0.071	0.021	-0.167
Decision-making time	-0.332	0.303	-0.030	0.602*
BIS (HC)	0.134	0.056	0.129	-0.222
**BART (all subjects)**				
Total score	-0.039	-0.074	0.012	-0.150
Number of burst balloons	0.312*	0.217	0.140	-0.128
Averaged pumps	0.127	0.034	0.114	0.042
Decision-making time	-0.186	0.063	0.118	0.301*
BIS (all subjects)	0.303*	0.276*	-0.006	-0.300

The data were correlation coefficient, *P<0.05. BART, balloon analogue risk task; BIS, Barratt impulsiveness scale.

**Figure 2:**
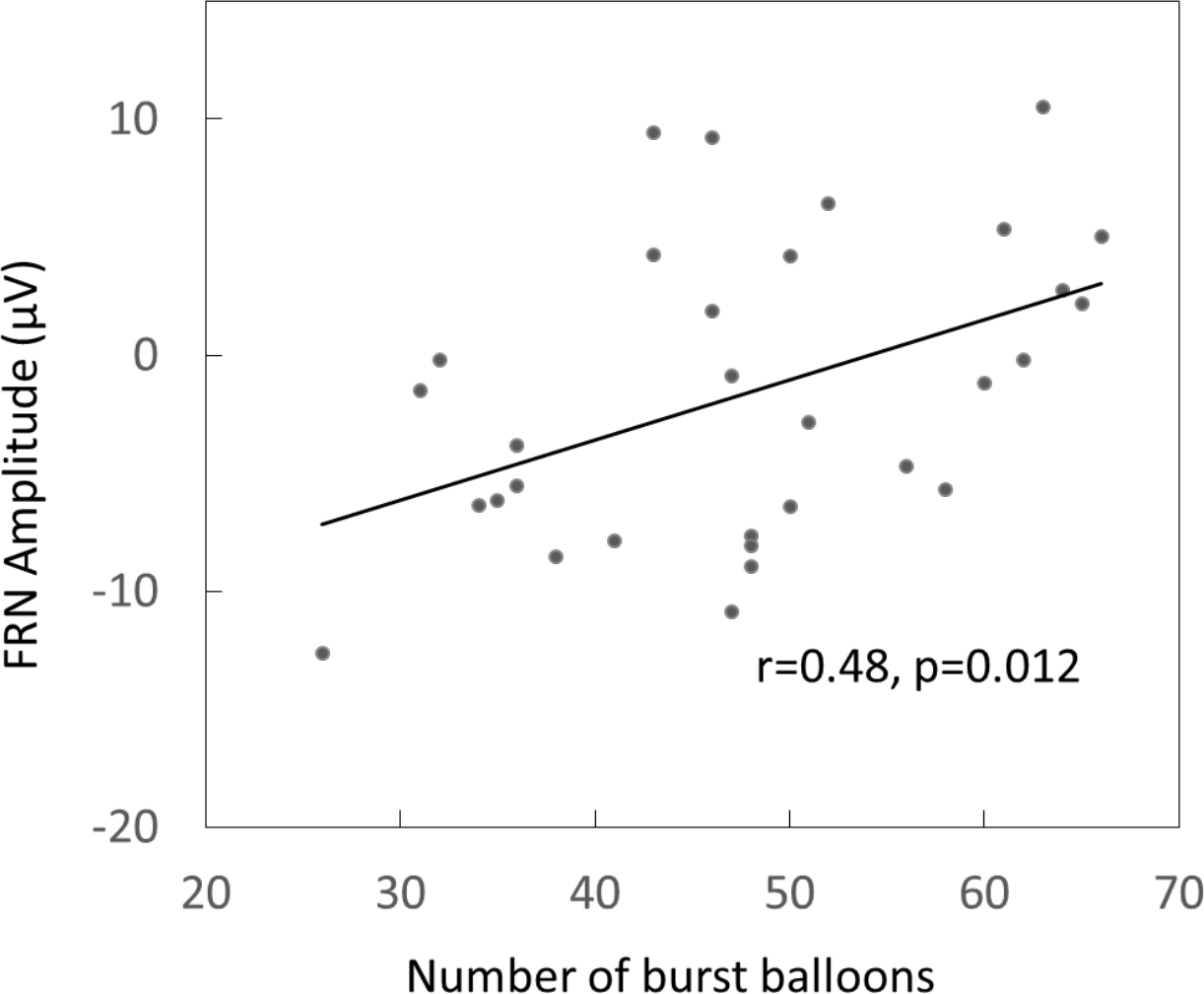
The Pearson correlation between the amplitudes of feedback-related negativity (FRN) and number of burst balloon in MA users.

But there was only significant correlation between the averaged peak FRN amplitude within frontal-central electrodes and the total burst balloons (r=0.48, P=0.012) in BART in the MA group, and this effect was still significant when the response time of BART (r=0.439, p=0.015) or the total BIS score (P=0.428, P=0.018) was controlled (**Figure 2**).

## Discussion

The study found that MA chronic users showed smaller FRN amplitudes in frontal-central regions compared to healthy controls in a risky decision-making paradigm, furthermore, these smaller FRN (representing less neural response to unfavorable feedback) for MA users were significantly related to higher number of burst balloons in the risky decision-making task. Besides, the study also identified MA users showed longer FRN latency compared to healthy controls, which correlated with the higher BIS scores. These results indicated that MA chronic users showed decreased neural sensitivity to negative feedback in fronto-central electrodes, which mimic MA users' real-world insensitivity to negative consequences from drug-using and preference to pursuit immediate reward, in line with reinforcement learning theory (6, 10, 12, 34). These results provide evidence for the basis of the neural mechanism of the risk decision-making among MA users.

The studies indicated that MA users showed a trend of taking more risky behaviors in the risky decision-making processes, which is consistent with previous studies. Individuals with substance dependence often showed risky and immediate reward preference over a relatively safe and “boring” option, especially when individuals faced the uncertain probability of different options they tend to put more attention on a potential gain but not a loss. This risky decision-making pattern was also found in chronic MA users by other risk-taking measurements, such as the Cups tasks, probabilistic feedback expectancy task, Iowa Gambling Task (3, 19, 35), which indicated that MA users' risk preference and reward sensitivity. Deficits in risky decision-making could be related to vulnerability toward the development of dependence, or long-term MA neurotoxicity, or negative consequences of drug use and bad psychosocial wellbeing (6, 9). There need more longitudinal studies to know better about the risky decision-making deficits that could be a potential important behavior feature of MA dependence.

The study found that the MA users showed the weakened and delayed FRN within frontal-central electrodes compared with healthy controls, and weakened FRN amplitudes significant related with more risk-taking behaviors. The FRN amplitudes were related to the subjective and behavioral indexes of reward sensitivity, the weaken FRN to unfavorable feedback in MA users reflected the poorer and slower neural responses compared with healthy controls. This was consistent with the findings of previous studies that less neural responses to the negative feedback existed in the patients with alcohol dependence, problematic gambling, or problematic Internet use (21–23). One study found that the female MA users had enhanced FRN after feedback in a simple gambling task compared with healthy controls, but the enhanced neural response to feedback was the extended impact from the anticipatory reward effect possibly (20). However, our study more focused on the neural responses to feedback and both female and male participants were involved. Although the variability of the FRN amplitude could be a potential biomarker representing poor decision-making ability for dependence, more studies about the brain potential changes during risky decision processes are needed.

No significant difference in the amplitude of P300 between MA users and healthy controls that demonstrated that there was no difference in later cognitive processing in risky decision-making. It implied that the ability of MA users to subsequently assign sufficient attention to motivationally salient events may not be impaired (36). But some previous studies found the blunted P300 after the feedback in the groups of problematic Internet users, alcohol users (21–23). The MA users in this study had averaged nearly 3 months abstinence from drug use might impact the results of P300 after feedback. However, the latency of P300 in MA groups was shorter than that in Healthy controls. However, the P300 latency of MA groups was shorter than that in healthy controls. Further analysis revealed that there were positive relationships between P300 latency and decision-making time (time period between feedback and implementing the decision) in Healthy controls and all subjects. It implied that MA users appeared earlier information processing in the late-stage of risky decision-making, while more studies are needed to explore the relationship between the P300 latency and the behavior of rushing into the implementation of a decision in MA users. These results of FRN and P300 suggest that MA users showed an insensitive neural response to feedback in early risky decision-making (FRN) but not late-stage (P300).

Maladaptive decision-making of chronic MA users may reflect neural or circuit-level dysfunction. Some studies revealed that FRN was most likely generated from the anterior cingulate cortex (ACC) when individual get external unfavorable feedback, and then ACC used the signal to modify the continuous performance (10). Moreover, some studies found that there were abnormal brain activation in the anterior cingulate, insula, right dorsolateral prefrontal cortex, ventral striatum to negative feedback when chronic MA users performed the risk decision-making tasks, which led to high risk-taking behavior even through negative consequences from the long-term drug use (4, 5). The previous study proved that there was the down-regulation of the dopaminergic system in the striatum in MA users, which was associated with the neurotoxicity and the cognitive deficits from MA use (37). In the future study, it is a good way to use methods of the brain electroencephalography and functional MRI to figure out what happens during the series decision-making.

And this study also found chronic MA users showed high impulsive personal traits, which significant with poor and late neural responses to negative feedback during risky decision-making, which indicated the individuals with higher impulsivity tended to rush into a decision while their neural response to negative consequences is a slow processing model (22, 38, 39). It was consistent with previous studies that high impulsive personality trait was related to risky decision-making and feedback processing (24). Previous studies showed that executive function deficits or impulsive personality traits were endophenotypes associated with amphetamine dependence (28). The impulsive decision-making pattern and cognitive impairments of MA users could be a premorbid factor or effects from drug neurotoxicity.

Some previous studies showed that some cognitive deficits of chronic MA users could partially recover after abstinence, but this cross-sectional study was hard to explain the dynamic changes of risky decision-making deficits over abstinence (31). Due to the regulations in the compulsory rehabilitation center, we just use the virtual reward on the risky decision task. It is conceivable that providing a real monetary reward for good performance may strongly influence risk-taking. So in the future study, the real money could be considered in the risky decision task. In this study MA users were abstinent for about 2 months, considering the possibility of dynamic changes of cognitive deficits after abstinence, it would be a benefit to recruit current MA dependent individuals in the future study.

## Conclusion

Our findings demonstrate that compared to healthy controls chronic MA users showed high impulsive trait and a trend of more risky behaviors which related with smaller Feedback Related Negativity (FRN) amplitudes in fronto-central electrodes in a risky decision-making task. The study suggests that chronic MA users show the features of the insensitivity to negative feedback, and blunted FRN could be a promising biomarker of the risky decision-making or impulsive decision-making pattern for the therapy or prevention of MA dependence.

## Data Availability Statement

The raw data supporting the conclusions of this article will be made available by the authors, without undue reservation.

## Ethics Statement

The studies involving human participants were reviewed and approved by Shanghai Mental Health Center (permission number: 2016-11R). The patients/participants provided their written informed consent to participate in this study.

## Author Contributions

NZ designed the study and analyzed and drew the draft version of the manuscript. TC and YZ recruited and collected the data. HS, XR and XL helped with collecting the data. HT and HJ helped data analysis. JD and MZ designed the study and contributed to the final version of the manuscript.

## Funding

The study was supported by the National Key Research-and-Development Program of China (2017YFC1310400), the National Nature Science Foundation (81501148, 81801319, 81771436), the Shanghai Municipal Health Commission (2018YQ45, 20184Y0134, 2017ZZ02021), and the Shanghai Mental Health Center (CRC2018YB02, 2014-QH-01).

## Conflict of Interest

The authors declare that the research was conducted in the absence of any commercial or financial relationships that could be construed as a potential conflict of interest.
